# Sex differences in prevalence and clinical correlates of internet addiction among Chinese adolescents with schizophrenia

**DOI:** 10.1186/s12888-024-05691-6

**Published:** 2024-04-05

**Authors:** Yunhui Zhong, Shuixiu Lai, Anquan Hu, Yuanping Liao, Yibo Li, Zheng Zhang, Xiang-Yang Zhang

**Affiliations:** 1 The Third People’s Hospital of Ganzhou, Ganzhou, China; 2 Jiangxi Environmental Engineering Vocational College, Ganzhou, China; 3 Institute of Psychology, Tianjin Medical University, Tianjin, China; 4 School of Education Science, Anhui Normal University, Wuhu, China; 5 CAS Key Laboratory of Mental Health, Institute of Psychology, Chinese Academy of Sciences, Beijing, China; 6 Department of Psychology, University of Chinese Academy of Sciences, 16 Lincui Road, Hai-Dian District, 100101 Beijing, China

**Keywords:** Sex differences, Internet addiction, Adolescents, Schizophrenia

## Abstract

**Background:**

Patients with schizophrenia (SCZ) exhibit sex differences in various aspects, and patients with SCZ have a high prevalence of internet addiction (IA). However, sex differences in IA among patients with SCZ mostly remain unstudied, particularly in Chinese adolescent patients with SCZ. This study investigated sex differences in prevalence, risk factors, and clinical correlates of IA among Chinese adolescent patients with SCZ.

**Methods:**

A total of 706 adolescent patients with SCZ were enrolled in this study using a cross-sectional design and a convenience sampling method. Demographics and clinical data of the patients were collected using a standardized clinical assessment form. The Positive and Negative Syndrome Scale (PANSS) and the Young’s Internet Addiction Test were used to evaluate psychopathological symptoms and IA respectively.

**Results:**

Overall, the prevalence of IA among Chinese adolescent patients with SCZ was 26.30% (95% CI: 23.09-29.60%). In Chinese adolescents with SCZ, there was a sex difference in the comorbidity of IA (males: 33.33% vs. females: 21.69%). Binary logistic regression analysis showed that IA was significantly predicted by good socioeconomic status in male and female patients with SCZ. City of living and PANSS total score were associated with IA in male patients with SCZ. In contrast, hospitalization rate and depression score were associated with IA in female patients with SCZ.

**Conclusion:**

Our study suggests sex differences in clinical correlates of IA in Chinese adolescent patients with SCZ. An additional longitudinal study is required to confirm the findings of the present study.

## Introduction

Schizophrenia (SCZ) is a severe psychiatric disorder that manifests in several ways, including delusions, hallucinations, disturbed thought processes, cognitive dysfunction, and impairment of social behavior [1]. SCZ is linked to a considerable economic burden, with annual expenses in the United States estimated at over $150 billion [2]. The lifetime prevalence of SCZ is approximately 1% of the world’s population, according to a systematic review [3]. A lifetime prevalence of 0.6–0.8% for SCZ has been reported in epidemiological studies conducted in China [4, 5]. SCZ is 50 times less prevalent in adolescents than it is in adults [6]. Adolescent patients with SCZ are characterized by prominent negative symptoms and disorganization [7, 8], have a poorer prognosis, and have a poorer response to treatment compared to adult patients with SCZ [9]. Early epidemiological studies revealed a male-to-female ratio between 1.4:1 and 2.2:1 for adolescent-onset SCZ [10].

In previous studies, sex differences in the demographics and clinical characteristics of patients with SCZ were observed [11]. Males typically present at a younger age of onset [12], have poorer premorbid functioning [13], have a poorer prognosis [14], and display more negative symptoms, according to most studies [15]. In contrast, women respond better to treatment with conventional antipsychotic medications, have a better short- and intermediate-term course, and exhibit more affective symptoms, hallucinations, and persecutory delusions [16]. Several biopsychosocial approaches, including genetic, susceptibility, and neurodevelopmental differences, have been proposed to explain the sex differences [17]. Additionally, earlier research demonstrated sex differences in adolescent patients with SCZ pathogenesis and prognosis, with outcomes quite comparable to those of adult patients [18, 19]. For example, males were shown to adhere to treatment better among adolescents with patients with SCZ, although females did better regarding social cognition [20].

According to studies, patients with SCZ have a high prevalence of addiction [21, 22], which significantly worsens their overall clinical course. For example, 50% of patients with SCZ abuse cannabis, compared to 1% of the general population; 29% of patients with SCZ abuse nicotine, compared to 13% of the general population, and 43–65% of patients with SCZ abuse alcohol, compared to 5% of the general population [23]. Internet use, including internet addiction (IA) in patients with SCZ, has recently attracted the attention of an increasing number of researchers [24, 25]. IA, also known as psychological dependence on the internet, has been linked to co-morbid forms of psychosis and is characterized by increasing resource investment in internet-related activities, unpleasant feelings when offline, increasing tolerance for the effects of internet access, and denial of problematic behaviors [26, 27]. According to a recent study of Korean patients with SCZ, which found a 22% prevalence of IA, men are more likely to have co-morbid IA [24].

To the best of our knowledge, no studies have looked at sex differences in IA in Chinese adolescent patients with SCZ. This study aimed to investigate potential sex differences in IA prevalence among Chinese adolescent patients with SCZ. As a secondary objective, we checked whether sex differences in IA in Chinese adolescent patients with SCZ were associated with specific clinical features and symptoms.

## Methods

### Procedure and sample

This study was carried out on 706 adolescent patients with SCZ at the Third People’s Hospital of Ganzhou, a public psychiatric hospital in Ganzhou, China. This hospital contains > 1400 inpatient beds and over 450 outpatients per day. In Ganzhou and its neighboring districts, which have a total population of almost 9 million, this hospital offers psychiatric services to patients and specializes in treating a variety of patients with psychiatric disorders. We reached every inpatient at the hospital using a cross-sectional naturalistic design. Data were collected from June 2018 to October 2021.

Inclusion criteria included: (1) Chinese Han patients between 13 and 18 years; (2) met the diagnosis of SCZ by two psychiatrists according to the Chinese version of Structured Clinical Interview for Diagnostic Manual of Mental Disorders-IV; and (3) were taking a fixed dosage of medication.

Exclusion criteria included: (1) having a serious physical disease; (2) abusing or being dependent on drugs and alcohol in addition to nicotine; and (3) not being able to provide written informed consent.

The Institutional Review Board of the Third People’s Hospital of Ganzhou approved our study protocol. All participants, or their legal guardians, gave their written informed consent after thoroughly explaining the study to each of them.

### Demographics and clinical characteristics

We collected demographic and clinical variables, including age, sex, education, family structure, socioeconomic status, place of residence, age of onset, age at first hospitalization, number of hospitalizations, and duration of illness from all participants. Overweight and obesity were determined using the gender-and age-specific body mass index cutoff points recommended by the Working Group for Obesity in China [28].

The Positive and Negative Syndrome Scale (PANSS) [29], which is clinically frequently used in patients with SCZ, was used to evaluate the clinical psychiatric assessment of the patients. Two psychiatrists participated in a PANSS training course to confirm the reliability and consistency of the scores. After several evaluations, the interobserver correlation coefficient for the PANSS total score remained above 0.8. Additionally, several earlier research has demonstrated that the 5-factor models of PANSS can accurately reflect the validity of clinical symptom dimensions [30]. We used the PANSS 5-factor model in the present study, which consists of the positive factor (P1, P3, P5, and G9), negative factor (N1, N2, N3, N4, N6, and G7), cognitive factor (P2, N5, and G11), excitement factor (P4, P7, G8, and G14), and depressive factor (G2, G3, and G6) [31].

The Chinese version of the IA test (IAT) [32, 33], frequently used to assess the presence and severity of IA, was used to measure IA. IAT is a 20-item self-report scale with three subscales: withdrawal and social problems, time management and performance, and reality substitution. Each item is scored on a 5-point scale, where 1 is “never” and 5 is “always.” Scores range from 20 to 100. This study defined IA was defined as having a score of 50 or more [34], which had been used in previous studies [35, 36]. In this study, Cronbach’s α for the IAT was 0.98.

### Statistical analyses

Descriptive statistics were performed for all measurements, with continuous variables expressed as mean ± standard deviation and categorical variables expressed as numbers and percentages. The normality of the variable distribution was determined using the Kolmogorov-Smirnov test. Given that all continuous data had a normal distribution, Chi-square tests for categorical variables and t-tests for continuous variables were used to compare the demographic and clinical characteristics of males and females. Additionally, separate analyses of variance for the boys and girls were carried out to examine the differences in clinical characteristics between subclinical groups with and without IA. Finally, binary logistic regression analysis was used to identify the elements that significantly influenced IA in males and females. Multiple comparisons were taken into account using Bonferroni corrections.

All data were analyzed using Statistical Package for Social Sciences (SPSS) 21.0 (SPSS Inc., Chicago, USA). *P* < 0.05 (two-tailed test) was considered statistically significant.

## Results

### Demographics and clinical characteristics of participants

A total of 706 inpatients participated in our study, including 282 males and 424 females. The mean age of the patients was 15.41 ± 1.45 years, ranging from 13 to 18 years. The mean duration of education was 8.48 ± 1.38 years, ranging from 6 to 12 years. The mean age of onset was 13.84 ± 2.14 years, ranging from 6 to 18 years. The mean duration of illness was 20.81 ± 23.12 months, ranging from 1 to 240 months. The mean age of hospitalization at onset was 14.45 ± 1.69 years, ranging from 9 to 18 years. The mean number of hospitalizations was 3.10 ± 2.19, ranging from 1 to 17.

The mean PANSS scores were: positive, 14.82 ± 6.83; negative, 23.93 ± 8.40; concrete, 5.06 ± 1.65; depression, 8.94 ± 4.20; excited, 13.39 ± 7.62; and total score, 98.06 ± 15.88.

### Sex differences in demographics and clinical characteristics in adolescent patients with SCZ

As shown in Table 1, all variables between male and female patients were significantly different (*p* < 0.05), except for obesity and PANSS total score. Female patients were younger; more educated; had a younger age of onset; had longer illness durations; younger hospitalization ages at onset; more frequent hospitalizations; higher positive, negative, and concrete scores; lower depression and excitement scores. The female patients were also more inclined to live with their parents, have siblings, have no family history, have poor socioeconomic status, and live in rural areas than male patients.

**Table 1:** Sex difference: demographics and clinical characteristics among adolescents with schizophrenia

Characteristics	Total patients	Males	Females	t/X^2^	P-value
	706	(*n* = 282, 39.94%)	(*n* = 424, 60.06%)		
**Family structure**					
Living with parents	668 (94.6)	260 (92.19)	408 (96.23)	5.395	0.020
Single parent family	38 (5.4)	22 (7.81)	16 (3.77)		
**Being the only child**					
Yes	150 (21.2)	92 (32.62)	58 (13.68)	36.328	0.000
No	556 (78.8)	190 (67.38)	366 (86.32)		
**Family history**					
Yes	114 (16.1)	72 (25.53)	42 (9.91)	30.542	0.000
No	592 (83.9)	210 (74.47)	382 (90.09)		
**Socioeconomic status**					
Good	512 (72.5)	232 (82.27)	280 (66.04)	22.391	0.000
Bad	194 (27.5)	50 (17.73)	144 (33.96)		
**Residence**					
Urban	178 (25.2)	94 (33.33)	84 (19.81)	16.423	0.000
Rural	528 (74.8)	188 (66.67)	340 (80.19)		
**Weight**					
Normal weight	468 (66.3)	196 (69.50)	272 (64.16)	2.195	0.334
Overweight	162 (22.9)	58 (20.57)	104 (24.53)		
Obese	76 (10.8)	28 (9.93)	48 (11.31)		
Age (years)	15.411 (± 1.455)	15.589 (± 1.574)	15.293 (± 1.358)	2.662	0.008
Education (years)	8.484 (± 1.381)	8.695 (± 1.631)	8.344 (± 1.167)	3.329	0.001
Age at onset (years)	13.839 (± 2.141)	14.326 (± 2.444)	13.514 (± 1.844)	5.023	0.000
Duration of illness (months)	20.810 (± 23.141)	16.901 (± 20.618)	23.410 (± 24.329)	–3.696	0.000
Age of hospitalization at onset (years)	14.450 (± 1.688)	14.745 (± 1.869)	14.255 (± 1.526)	3.816	0.000
Hospitalization numbers	3.099 (± 2.187)	2.653 (± 2.520)	3.396 (± 1.880)	–4.489	0.000
Positive subscore	14.824 (± 6.833)	13.759 (± 7.180)	15.533 (± 6.500)	–3.406	0.001
Negative subscore	23.932 (± 8.406)	22.340 (± 8.130)	24.991 (± 8.420)	–4.153	0.000
Concrete subscore	5.060 (± 1.648)	4.887 (± 1.928)	5.174 (± 1.420)	–0.283	0.023
Depressive subscore	8.943 (± 4.203)	10.192 (± 4.143)	8.113 (± 4.034)	6.633	0.000
Excited subscore	13.391 (± 7.628)	16.348 (± 7.500)	11.424 (± 7.057)	8.854	0.000
PANSS total score	98.068 (± 15.880)	97.702 (± 21.837)	98.311 (± 13.455)	–0.499	0.618

Values expressed as number (%) or mean (± standard deviation)

*P < 0.05, **P < 0.01

### Sex differences in the prevalence of IA in adolescent patients with SCZ

IA affected 26.3% (186/706) of the population. Females (21.69%, 92/424) were less likely to have a comorbid IA compared to males (33.33%, 94/282; χ^2^ = 11.82, df = 1, *p* < 0.001). Males had an IA rate that was 1.80 times higher than females (B = 0.59, Wald statistic = 11.674, *p* < 0.001, odd ratio (OR) = 1.804, 95%CI = 1.286–2.531).

### Factors associated with IA in male adolescent patients with SCZ

In males (*n* = 282), there were 94 (33.33%) and 188 (66.67%) patients with and without IA, respectively. Male patients with and without IA had significant differences among them in terms of age (15.17 ± 1.23 vs. 15.79 ± 1.68, F = 26.009, *p* < 0.001), duration of disease (23.47 ± 29.61 vs. 13.61 ± 13.05, F = 79.740, *p* < 0.001), age at onset (13.36 ± 3.02 vs. 14.80 ± 1.93, F = 21.229, *p* < 0.001), number of hospitalizations (3.21 ± 3.21 vs. 2.37 ± 2.04, F = 11.952, *p* < 0.01), depression score (11.11 ± 5.56 vs. 9.73 ± 3.12, F = 38.844, *p* < 0.001), PANSS total score (107.38 ± 21.31 vs. 92.86 ± 15.58, F = 24.194, *p* < 0.001), good socioeconomic status (91.49% vs. 77.66%, χ^2^ = 8.217, df = 1, *p* < 0.01), and living in urban areas (57.45% vs. 21.28%, χ^2^ = 36.894, df = 1, *p* < 0.001). These differences continued to be significant after the Bonferroni correction (*p* < 0.01). ICorrelations between the IA total score and age, age at onset, duration of illness, age of hospitalization at onset, number of hospitalizations, socioeconomic status, place of residence, negative score, depressive score, excited score, and PANSS total score were statistically significant (Table 2). Furthermore, binary logistic regression revealed that socioeconomic status (good) (OR = 8.945, 95%CI: 2.495–32.073, *p* < 0.001), place of residence (urban) (OR = 4.294, 95%CI: 2.201–8.376, *p* < 0.001), and PANSS total score (OR = 0.944, 95%CI: 0.923–0.966, *p* < 0.001) were risk factors for IA in this group of adolescent patients with SCZ (Table 3).

**Table 2:** Correlation between internet addiction and demographic data and clinical characteristics in male and female patients

Characteristics	Males (*n* = 282)	Females (*n* = 424)
Age (years)	–0.121*	0.107*
Age at onset (years)	–0.267**	0.268**
Duration of illness (months)	0.258**	–0.197**
Being the only child	0.061	–0.136**
Age of hospitalization at onset (years)	–0.143*	0.154**
Hospitalization numbers	0.152*	–0.115*
Socioeconomic status	–0.145*	–0.331**
Residence	–0.333**	–0.139**
Weight	0.022	0.079
Positive subscore	–0.093	0.274**
Negative subscore	0.244**	–0.395**
Concrete subscore	–0.047	–0.247
Depressive subscore	0.201**	0.386**
Excited subscore	0.417**	0.428**
PANSS total	0.279**	0.081

*P < 0.05, **P < 0.01

**Table 3:** Binary logistic regression analyses of the determinants of internet addiction in male patients

Variables	B	Wald X^2^(df = 1)	P-value	OR	95% CI
Age	0.375	0.616	0.433	1.455	0.570–3.714
Duration of illness	–0.059	2.081	0.149	0.943	0.870–1.021
Age at onset	–0.050	0.012	0.913	0.951	0.383–2.363
Hospitalization numbers	0.050	0.411	0.521	1.051	0.902–1.225
Socioeconomic status, good	2.191	11.312	0.001	8.945	2.495–32.073
Residence, urban	1.457	18.276	0.000	4.294	2.201–8.376
Depressive score	0.011	0.054	0.816	1.011	0.920–1.111
PANSS total score	–0.057	24.138	0.000	0.944	0.923–0.966

CI, confidence interval; OR, odds ratio

### Factors associated with IA in female adolescent patients with SCZ

In females (*n* = 424), there were 92 (21.70%) and 332 (78.30%) patients with and without IA, respectively. Female patients with and without IA had significant differences among them in terms of age (16.57 ± 1.55 vs. 15.22 ± 1.29, F = 15.927, *p* < 0.001), age of hospitalization at onset (14.54 ± 1.62 vs. 14.17 ± 1.49, F = 5.588, *p* < 0.05), number of hospitalizations (3.30 ± 1.54 vs. 3.42 ± 1.96, F = 28.248, *p* < 0.001), positive score (17.70 ± 6.46 vs. 14.93 ± 6.39, F = 4.240, *p* < 0.05), depression score (10.61 ± 3.42 vs. 7.42 ± 3.92, F = 8.184, *p* < 0.01), PANSS total score (99.39 ± 15.52 vs. 98.01 ± 12.84, F = 9.029, *p* < 0.01), only child (21.74% vs. 11.42%, χ^2^ = 6.464, df = 1, *p* < 0.05), good socioeconomic status (91.30% vs. 59.04%, χ^2^ = 33.444, df = 1, *p* < 0.001), and living in urban areas (30.43% vs. 16.87%, χ^2^ = 8.347, df = 1, *p* < 0.01). These differences continued to be significant after Bonferroni corrections (*p* < 0.01). Correlations between the IA total score and age, age at onset, duration of disease, an only child, age of hospitalization at onset, number of hospitalizations, socioeconomic status, place of residence, positive score, negative score, depression score, and excited score were statistically significant (Table 2). Furthermore, the binary logistic regression revealed that socioeconomic status (good) (OR = 4.242, 95%CI: 1.661–10.836, *p* < 0.01), number of hospitalizations (OR = 0.673, 95%CI: 0.547–0.828, *p* < 0.001), and depression score (OR = 0.819, 95%CI: 0.744–0.902, *p* < 0.001) were risk factors for IA in this group of adolescent patients with SCZ (Table 4).

**Table 4:** Binary logistic regression analyses of the determinants of internet addiction in female patients

Variables	B	Wald X^2^(df = 1)	P-value	OR	95% CI
Age	0.347	3.446	0.063	1.415	0.981–2.042
Age of hospitalization at onset	–0.382	5.117	0.054	0.683	0.491–0.950
Hospitalization numbers	–0.396	14.054	0.000	0.673	0.547–0.828
Being the only child, yes	0.411	1.450	0.228	1.509	0.772–2.947
Socioeconomic status, good	1.445	9.120	0.003	4.242	1.661–10.836
Residence, urban	0.015	0.002	0.963	1.015	0.536–1.922
Positive score	–0.011	0.171	0.679	0.989	0.939–1.042
Depressive score	–0.199	16.550	0.000	0.819	0.744–0.902
PANSS total score	0.021	3.174	0.075	1.021	0.998–1.045

CI, confidence interval; OR, odds ratio

## Discussion

To the best of our knowledge, this is the first significant clinical investigation to examine sex differences in comorbid IA in Chinese adolescent patients with SCZ. Our key findings are as follows: (1) there was a 26.3% prevalence of IA among Chinese adolescent patients with SCZ, with a higher prevalence of IA in males (33.33%) than in females (21.69%); (2) good socioeconomic status was the strongest predictor of IA for both sexes; (3) for males with SCZ, living in a city and having a high PANSS total score were significantly associated with IA, whereas for females with SCZ, the number of hospitalizations and depression scores were significantly associated with IA.

Studies of culturally diverse psychiatric or general populations in the prevalence of IA have been examined, but the findings have not been conclusive. Based on the IAT, Lam, Peng [32] found that 10.8% of Chinese adolescents aged 13–18 year were moderately to severely addicted to the Internet. A meta-analysis of data based on 70 studies covering 122,454 Chinese adolescents found an overall prevalence of IA of 11.3% [37]. Notably, the prevalence of IA among adolescents with schizophrenia in our study was 26.3%, indicating a relatively high prevalence of IA compared to the prevalence in the general adolescent population in China. Schizophrenia is a well-known risk factor for addictive disorders [38]. Brunette, Mueser [39] showed that more than half of the patients with first-episode psychosis had a substance use disorders. Also, Desai and Potenza [40] found that individuals with schizophrenia/schizoaffective disorder may be at particularly high risk for problem and pathological gambling. Taken together, these findings suggest that higher prevalence of IA may occur in patients with schizophrenia, especially in adolescents with schizophrenia.

Most studies have discovered that men often have a higher comorbidity of IA. A study by Chen et al. [41] with a reasonable sample size (*n* = 5249) indicated that males (12.3%) were about 2.5 times more likely than females (4.9%) to have IA. The prevalence of IA was reported to be 26.39% in males and 16.95% in females in a Korean study of patients with SCZ (*n* = 368) [24]. There are no significant sex differences in psychiatric patients with comorbid IA, according to some studies. For example, no sex differences were found between male and female adolescents in a cross-sectional population-based epidemiological study of Turkish adolescents with psychosis (*n* = 310) [42]. Our study revealed a higher prevalence of IA in male adolescents with SCZ than in female adolescents, which is consistent with most previous studies focusing on adult SCZ [24] or the general adolescent population, even after adjusting for potential confounders [37, 43, 44].

The relatively high prevalence of IA in male adolescent patients with SCZ in our cohort has several potential reasons. First, research has shown that male adolescents are more likely to have access to the internet and engage in online activities like gaming, pornography, and gambling, which can result in compulsive internet use [41, 45]. Second, females with SCZ prefer to connect with others in the real world because they have fewer social cognitive impairments than males with SCZ [20, 46, 47]. Finally, according to Willhite et al., males with psychotic disorders report less positive social support than their female peers and perceive that they receive slightly more criticism [48]. Male adolescents lack adequate social support, feel more isolated, and develop an internet addiction [49].

Furthermore, this study discovered that good socioeconomic status among male and female adolescents with SCZ was the strongest indicator. Similar findings have been drawn from other studies [37, 50]. Families with bad socioeconomic status were thought to have more children, a lower household income, only the father working, and inadequate environmental conditions and opportunities, according to Kayri et al. [50]. Adolescents with good socioeconomic status have more internet access than those with bad socioeconomic status due to these causes.

Our study discovered clinical characteristics unique to male and female adolescent patients with SCZ and socioeconomic status. The current study discovered that IA in male adolescents with SCZ was significantly associated with urban residence and lower PANSS scores. Adolescents living in urban areas are said to be more likely than those in rural areas to have access to the internet via cell phones and computers [51]. Higher PANSS scores are generally thought to indicate a poorer condition for patients, such as attention deficit or delusional interpretations and motivational deficits that result in rejection of internet use [52].

However, we found that IA was associated with fewer hospitalizations and higher depression scores in female IA patients. In our study, the cell phones of patients were kept in custody by the hospital staff and were denied access to the internet. Fewer hospitalizations indicated more opportunities to have a cell phone or computer to access the internet. We are well aware that a loss of interest or enjoyment in practically all activities is one of the key signs of depression [53]. We believe that adolescents who are depressed to a high degree are not interested in the internet. Notably, earlier research has found a positive association between depression and IA [54, 55]. One explanation is that depression is measured indirectly using the PANSS scale. Future studies should use scales to measure depression specifically.

The current study has several limitations that should be considered. First, we only collected a few demographic and clinical variables, including daily internet usage, the type of internet use, and medical conditions. Second, because the study was cross-sectional, it could not draw any causal inferences. Third, the only patients with SCZ in our sample were adolescents hospitalized in Ganzhou, China. Therefore, the findings of this study cannot be generalized to all adolescent patients with SCZ. Finally, we did not categorize IA into specific subtypes in our study. Therefore, there may be differences in the susceptibility of male and female patients with SCZ to IA.

## Conclusion

In conclusion, our research indicates that IA is widespread among Chinese adolescent patients with SCZ, both male and female. IA has a negative impact. Hence psychiatrists must check for IA in male and female patients with SCZ. Our findings suggest that gender-specific prevention and intervention techniques may reduce IA in Chinese adolescent patients with SCZ. Patients with good socioeconomic status should receive more attention, whether male or female. Additionally, the city of living and PANSS total score should receive special attention for males with SCZ. The number of hospitalization rates and depression scores for females with SCZ should be carefully monitored.

## Data Availability

The raw data supporting the conclusions of this article will be made available by the corresponding author if necessary.
